# Book Reviews

**Published:** 1833-07

**Authors:** 


### CRITICAL ANALYSES.

Quæ laudanda forent, et quæ culpanda, vicissim,

Illa prius, cretâ; mox hæc, carbone, *notamus.*—PERSIUS.

*Observations on the Testicles.* By JAMES RUSSELL, F.R.C.S., late Regius Professor of Clinical Surgery in the University of Edinburgh, Vice-President of the Royal Society of Edinburgh, &c.—Foolscap 8vo. pp. 276. Black, Edinburgh; Longman and Co., London.

NOTWITHSTANDING the self-gratulatory tone of the dedication, this volume is written in a plain, easy, and familiar style; and we have derived some pleasure from its perusal, which is more than we frequently do from elementary works of a preceptive character. The chief value of such works lies in their simplicity and clearness, and their most important duty is to inculcate established principles, and to reiterate truths acknowledged and confessed by all. Novelty is no recommendation to such works; for, although lateness of discovery does not lessen the worth of facts, the majority of truths are homely and old-fashioned. Hence, "Elements," "Lectures," and "Introductions," however useful they may prove to the student, do not in general do more than repeat to the veteran a thrice-told tale.

But these observations of Mr. RUSSELL hold a somewhat higher rank, and demand, of course, more general attention; for, being founded on lectures composed "about thirty years ago," and "improved by the additional information which the author has since acquired from experience and study," they cannot but contain some practical information worthy the attention of all; especially as we learn that the author has "had many opportunities, during the long period above mentioned," of studying the important class of affections about which he writes, "under a great variety of modifications."

The essay consists of seven chapters, which treat successively of, 1, *the number of the testicles;* 2, *descent of the testicles;* 3, *diseases of the scrotum;* 4, *tunica vaginalis;* 5, *hydrocele;* 6, *diseases of the testicles;* 7, *diseases of the spermatic cord.*

The first chapter commences with a truism, viz. that "a man is naturally provided with two testicles, which are lodged in the scrotum;" and it might be supposed that the thesis would exclude much else. But the author has contrived to collect details of some cases of monorchides and triorchides, which are pathologically and physiologically interesting.

"In a few individuals, the number of testicles has been found greater or less than the usual standard, more frequently less, and sometimes entirely wanting. The total want of testicles in the scrotum is a circumstance very alarming to parents, from the apprehensions which they naturally entertain respecting the virility of their child. These apprehensions, however, are for the most part groundless, as the absence of testicles from the scrotum arises merely from their having failed to descend from the abdomen before birth; there being undoubted instances of men without the vestige of testicles in the scrotum having become the fathers of numerous families. And in dissecting the bodies of persons of this peculiar formation of parts, one or two testicles, of a full size, and perfect in all respects, have almost invariably been found in the abdomen, so that a dissection in which the testicles were entirely wanting, both in the scrotum and in the abdomen, is a very rare occurrence.

"A few cases of monorchides of a somewhat equivocal character, are found in the records of medicine. They are modified by different circumstances, which constitute three varieties.

"In the first variety, the solitary testicle was divided in the middle by a deep fissure, the lobes on each side were as large as a full grown testicle, and each was provided with a spermatic chord, which ran up to the same side of the body. The fissure, the lobes, and the duplication of parts, obviously result from the partial coalescence of two testicles.

"The second variety has undoubtedly the same origin, only the coalescence is more general, and the incorporation more complete. The single testicle was much larger than in an ordinary full-sized testicle, equable in its surface, without any deep fissure dividing it into lobes, and provided with two spermatic chords, running to the different sides of the body. From the simple structure of the testicles, it is easily conceivable how both might be incorporated, without destroying their function as secreting organs.

"The third variety agrees with the other two, in having one testicle and two spermatic chords, while it differs from them in the circumstance of both spermatic chords running to the same side of the body. The origin of this variety is not so obvious, though, like the other two, it is not productive of any inconvenience to the individual." (P. 1.)

"The annals of medicine contain many cases of reputed triorchides, though it is not possible to lay down any general rules for the diagnosis, since, from the nature of the peculiarity, their true character can be ascertained only by the result of a special investigation. Various authors mention the peculiarity of three testicles as hereditary in certain families.

" Although there is no natural limit to the number of testicles, the existence of more than three is an exceedingly rare occurrence. There are, indeed, many cases recorded of persons with four testicles, but the fact has not been verified by dissection. It has been said that persons have appeared with five, or even six testicles. In the person who had six testicles, four were of the natural size, and two much smaller. I shall not, however, dwell on a discussion for which the data are too defective to lead to any positive conclusion.

" With regard to the amorous propensities and generative faculties of persons with supernumerary testicles, the report of authors is in general favorable to their being more powerful than in other men. But as those authors often indulge in a playsome humour, their evidence must be taken with a certain degree of reserve. For, in the investigation of such a subject, it is hardly philosophical to mention the case of a monk with three testicles, who was so salacious as to have indomitable passions, which prevented him from keeping his vow of chastity; or that of a land-grave, with a like peculiarity, who was allowed a concubine as a reasonable indulgence to a man of his amorous complexion, who could not remain satisfied with the use of a single woman."* (P. 7.)

We think our author places rather too implicit reliance upon some of the cases referred to as recorded in "the Annals of Medicine." That the testicles may be lobulated, and that, from peculiar circumstances, they may become united, will be found to be only in accordance with the established laws of epigenesis; but the occurrence of a third or fourth distinct testicle, (not a lobule, or lobules, detached or attached,) and especially the passage of the two spermatic cords to the same side, are such wide wanderings, as to require the most direct and circumstantial evidence to warrant their truth, and to convince the world that some errors in the examinations have not vitiated the accounts; especially as neither Morgagni, nor Haller, nor Meckel, ever discovered a third testicle in the dissections of reputed triorchides," although other authorities, of less weight, have put several supposed cases on record. These, however, have most probably been detached lobules, mistaken for distinct testicles.

With regard to the Descent of the Testicles, the subject of the second chapter, the author observes, that nothing is known respecting the cause of occasional retardation; and he continues,

" It is wholly unconnected with any imperfection in the confined testicle, since, upon an average of observations, the retained testicle

* " A dog, remarkable for his salacity, had two testicles in the scrotum, and one in the abdomen.

is as fully formed and as large as those which have descended into the scrotum." (P. 12.)

This is certainly contrary to our experience: we have never met with a case in which the descent of the testicle was delayed, in which its development was not retarded, if not entirely arrested. Cases may occur in which, from preternatural impediments in the canal, the descent may be occasionally delayed or prevented; but, in general, we believe that the want of development is closely connected with the persistence of the testicles in the abdomen.

The following practical remarks are good; reports of such cases of error we have occasionally published in our Journal.

"When a testicle is arrested in its progress through the inguinal canal, it produces a swelling in the groin, which is readily mistaken for an inguinal hernia. Both complaints occupy the same place in the inguinal canal, both proceed from the protrusion of a viscus from the abdomen, and, if the medical attendant be not aware that the patient has no testicle in the same side of the scrotum, he regards the case as an inguinal hernia. Under this mistake, a fruitless attempt is made to reduce the hernia, and, when this attempt fails, a bandage is applied, which, by exciting pain, leads to a more accurate examination of the symptoms, and to the subsequent discovery of the true nature of the case; for, when an arrested testicle is the cause of the swelling, there is greater sensibility to pressure, and a peculiar sensation which characterizes the feeling of a testicle. The difficulty of the diagnosis is occasionally increased, by the complication of two complaints, which the late descent of the testicle contributes much to favour. In the first place, when the patient has passed the age of puberty, the large size of the testicle widens the inguinal canal to a preternatural degree. In the second place, the surrounding parts have their disposition to contract which existed in early life greatly impaired, so that, from the concurrence of those two causes, there is an opening left into which some of the abdominal viscera may easily enter, and produce a hernia of the congenital form." (P. 12.)

After quoting the very curious case recorded by Mr. Hutcheson, in which a sailor had imposed upon the examining surgeons many times, by elevating his testicles into the inguinal passages, and thus simulating a double hernia, those curious deviations from the ordinary course of descent are referred to, in which the testicle, instead of descending through the inguinal canal, accompanies the femoral vessels in their progress under Poupart's ligament, making its appearance at the bend of the thigh. Arnaud gives several instances of this singular variety. The most instructive case is detailed at considerable length. The following are the chief points worthy attention.

"An officer, about forty years of age, consulted Mr. Arnaud respecting a swelling in the bend of the thigh, which was taken for a hernia. Upon an accurate examination of the case, however, Mr.

Arnaud satisfied himself that the swelling was not a hernia, but a misplaced testicle. He adduces three reasons in support of his opinion. 1st, That the officer had not a testicle in the same side of the scrotum. 2d, That the swelling had the form and consistence of a testicle, the appearance of the spermatic chord alone being sufficient to distinguish the case from a case of crural hernia. 3d, That pressure produced exactly the same sensation on this as on the other testicle." (P. 23.)

Our author gives to the most commonplace truths an interest, by the appositeness of his illustrations, so that we are tempted to multiply our extracts.

"The arrival of the testicles in the scrotum does not produce any change in the constitution. They are of a small size, and are not endowed with much sensibility during infancy; when full grown, they are rarely equal in size, a circumstance which ought to be known by all practical surgeons; otherwise their ignorance may lead to very distressing consequences. Fabricius Aquapendente gives a most instructive instance of this in the case of a young man, who, upon observing his testicles to be unequal in size, became alarmed, and consulted a rupture-doctor about his supposed disease. The quack pronounced the case to be very alarming, and advised the immediate extirpation of the testicle. The patient, however, being unwilling to submit to so severe an operation without farther advice, consulted Aquapendente, who relieved his fear, by satisfying him that the supposed disease was nothing more than a natural inequality in the size of the testicles, a difference which almost constantly takes place.

"The testicles likewise are in general suspended at unequal distances from the pubis." (P. 24.)

In the third chapter, on the Diseases of the Scrotum, there is much instructive matter, collected from various sources; but, as it chiefly consists of extracts from the works of Acrel, Titley, and others, it will not afford us many quotations: we will take, however, the following.

"The scrotum is more predisposed to mortify than most parts of the body. It occasionally mortifies at the termination of tedious exhausting fevers, and on attacks of erysipelas. Such cases are always severe, though not fatal, excepting under circumstances particularly unfavorable. The whole of the scrotum is sometimes completely destroyed, and afterwards completely regenerated, even to the production of the hair. This, however, very rarely occurs. Even the perfect regeneration of the skin is by no means a constant termination. In those cases which I have seen, the naked surface of the testicle, or of the tunica vaginalis, was, after the cure, covered only with a thin pellicle, which adhered to the subjacent parts, and did not possess any mobility. This pellicle or cicatrix is often so limited in extent, as to confine the testicles to one situation, and sometimes even to subject them to an inconvenient degree of pressure. In one case, the constriction was so great as to create the most excruciating pain, which rendered the life of the patient miserable, and induced him to submit to the removal of a testicle. But as this partial operation did not procure complete relief, he soon after requested to have the other testicle also removed. This was an extreme case. But when the tendency to constriction once begins there is no method known of arresting its progress, nor of palliating its effects." (P. 64.)

"The most singular disease of the scrotum is the growth of a tumor of enormous size. In a memorable case of the kind, Ger. Ephr. 1692, the tumor attained the weight of more than 200 lb., a weight considerably greater than the weight of a well grown man of ordinary stature. These tumors, in general, begin insensibly without pain, and are not perceived till they attract notice by an obvious swelling. In a few cases they are the consequences of a blow, or their commencement is marked by a slight attack of pain, which is temporary, and does not return during the course of the complaint. Their progress is gradual and regular, and they may often be traced back for fifteen or twenty years. They do not occasion any inconvenience, excepting what arises from their bulk and weight. They are not only free from pain, but endued with very low powers of sensibility, since, neither the application of caustic nor the introduction of setons, excite any troublesome degree of irritation. A friend of mine, who practised some time in the West Indies, informed me that the rats sometimes fed upon these enormous tumors, while the patient lay in a most helpless condition, and was unable to defend himself from their attacks. The tumors bore being handled with considerable roughness, without the patient suffering from this rude treatment, excepting when the pressure was made on the part of the surface corresponding to the situation of the testicle; then, indeed, the patient complained of pain, as the testicle still retained its natural sensibility, or even possessed it in an unusual degree. The growth of such immense swellings does not affect the constitution, nor produce any symptom of debility. It does not, in all cases, even impair the function of generation, as Delpech particularly mentions that neither the penis nor testicles had lost any thing of their natural faculties. In this respect, however, the symptoms are not uniform, since, in some cases, the functions of the testicles seem to be suspended or impaired. In the case mentioned by Mr. Corse Scott, the patient had not had any connexion with a female for ten years before the time Mr. Scott saw him. In the case described by Dr. Titley, the patient had lascivious desires and erections, but no emissions. While Dr. Wells states that the patient's health remained unimpaired, while his virile powers gradually diminished, as the scrotal tumor increased.

"It is necessary to investigate these particulars with great care, since the expediency of saving or of removing the testicle often depends upon the result of this investigation.

"This very singular disease of the scrotum belongs to the warmer climates of the globe, the East and West Indies, and the correspondent latitudes of Africa. It is endemic, and very prevalent among the Bambara nation, on the coast of Guinea, among whom the misfortune of having a monstrous testicle is regarded as a mark of nobility. When the patient goes out to ride, the testicle is supported on a bowl placed on the pummel of the saddle; and when of the largest size, supported on a sheet passed over the shoulders, and dragged along the ground, when he attempts to walk.

"I know of only two well authenticated cases of this disease having originated in Europe. One occurred in the practice of Mr. Liston, Surgeon to the Royal Infirmary, Edinburgh; and the other in that of Mr. Delpech, of Montpelier. There is a third case, by Mr. Hall, of Manchester, probably of the same kind, though, as the symptoms are not decidedly marked, I have not included it in the number of well authenticated cases." (P. 68.)

It appears, from Mr. Russell's account, that the chimney-sweeper's cancer is rarely met with in Edinburgh, although of such frequent occurrence in London; and, as he observes, from the Parisian surgeons being silent on the subject, it is probable that it seldom occurs in Paris.

The chapter on the Tunica Vaginalis is short, and will not afford a single extract; but the following, which treats of Hydrocele, and in which the diagnoses are canvassed, and the merits of the different modes of cure discussed, will afford us matter for several.

"A hydrocele may be mistaken for a disease of a very different nature, or, conversely, another disease mistaken for a hydrocele; or, although the case be actually a hydrocele, it may be accompanied with singular circumstances, which are not disclosed at the time of the investigation. Thus, a hydrocele retaining its transparency may, instead of containing a fluid, contain a collection of hydatids. This case, though rare, I have known to occur, to the great embarrassment of the operator. A still more rare instance of a case of transparency not being an absolute criterion of hydrocele is mentioned by Richter, in the case of a patient who had a rheumatic swelling of the testicles, in which the affected testicle was transparent. The affection passed from the one testicle to the other, an alternation characteristic of rheumatic complaints. Besides hydatids, I have known an adventitious encysted transparent tumor adhering to the epididymis, and filling part of the tunica vaginalis. The existence of such a tumor is not known previously to the operation.

"But the most frequent causes of difficulty arise from want of transparency, either from opacity in the contained fluid, or from the preternatural thickness and consequent opacity of the tunica vaginalis. This latter is by far the most frequent. It is likewise, in general, accompanied with a degree of firmness, which prevents the fluctuation of the contained fluid, or the peculiar sensation of the testicle from being indistinctly perceived. When the hydrocele is tense, and of long standing, it is frequently accompanied with occasional acute lancinating pains, resembling those of a scirrhous testicle. Under these imposing symptoms a hydrocele has been removed as a case of scirrhous testicle, when a subsequent dissection has shewn the testicle to have been quite sound, and castration unnecessary. So calamitous a mistake points out the expediency of, in all cases, dividing the tunica vaginalis before proceeding to operate." (P. 111.)

"Another cause of deception is presented by a singular modification of congenital hernia. In this case, the lower portion of the omentum in contact with the testicle became soft from mortification: this preternatural softness misled the surgeon, who, not being prepared to expect any such change, conceived the case to be a hydrocele. The mistake, however, did not occasion any serious inconvenience." (P. 117.)

"The tendency of a hydrocele to increase is not circumscribed within any determinate limits. It sometimes attains a most enormous bulk. Mr. Cline drew off six quarts of fluid from a hydrocele on the person of Mr. Gibbon, the celebrated historian. But by far the largest on record is one mentioned by Mursinna, which was twenty-seven inches in its long axis, and seventeen in its transverse. The enormous size of this hydrocele almost exceeds the bounds of credibility; I shall therefore give the measurement in the author's own words: 'Diese Geschwulst betrug in ihrer grossten Lange, von oben bis unten, drey Viertheil einer Elle, und in der Mitte, im Durchschnitt von einer Seite zur andern, eine halbe Elle weniger einem Zoll." (P. 124.)

We extract the following as being a good summary of the results of different modes of treatment pursued in cases of hydrocele, and as affording a view of the opinion arrived at by the author, after so long a practice and such considerable experience.

"Whatever may be the cause of the hydrocele, the particular case under consideration may be either idiopathic or symptomatic of some other affection. Mr. Pott relates a very instructive case, in which the hydrocele was evidently dependent on a fit of gout, as the swelling disappeared along with the departure of the gout. And Sir E. Home relates three cases symptomatic of an irritation in the urethra, in which the hydrocele disappeared upon the cure of the strictures.

"Hydrocele, though a troublesome complaint, and very annoying from its unwieldy bulk, is rarely painful, and never dangerous. It occurs at all periods of life. Infants are occasionally born with hydrocele; but in them, or in children at an early age, the hydrocele often admits of a spontaneous cure, or its cure may be promoted, and insured almost to certainty, by the application of stimulating embrocations. But hydrocele, in patients of advanced years, is a chronic, stationary complaint, which does not usually undergo any favorable change spontaneously. In a few cases, indeed, the accumulated fluid is completely removed by absorption.

"Besides a spontaneous cure of hydrocele by the natural powers of the system, an accidental cure is sometimes obtained by the rupture of the containing parts. Dr. Douglas relates two cases of tense hydroceles, in both of which the tunica vaginalis gave way upon a slight inflexion of the body. The effused fluid escaped into the surrounding cellular membrane, from which it was speedily absorbed. A like rupture is sometimes occasioned by external violence. In either case the cure is complete for a time, but not always permanent. In one case, in which the parts were greatly distended, not only the tunica vaginalis, but the integuments of the scrotum also gave way, in consequence of a great exertion. By this means the whole fluid was completely evacuated, and a permanent cure obtained.

"The very frequent occurrence of hydrocele has afforded ample opportunity to try various methods of cure, and to ascertain their respective values.

"The only mode of cure now employed consists in evacuating the fluid by an operation. This operation is more or less simple, according to the object which the surgeon has in view. If his sole object be to evacuate the fluid, without using any precaution to prevent a return of the collection, he has only to make an opening into the cavity of the tunica vaginalis, by which the fluid escapes. The mere evacuation of the fluid, however, produces only a temporary, or what is in general termed a palliative, cure. It is, however, so easy, and for a time relieves the patient so completely, that it is often employed as a matter of convenience. It is simple and easily performed; but may prove troublesome or dangerous, by imprudence or mismanagement. If the surgeon is not sufficiently on his guard, he may wound the testicle, or the artery of the spermatic cord, which, by occasioning an unrestrainable hæmorrhage, has led to the loss of the testicle; or if, by undervaluing the risk of irritation, an attack of inflammation has been excited subsequent to the operation, the consequences have proved fatal.

"When the surgeon has a higher object in view, by using means to prevent a relapse, the cure is termed radical. This object is attainable in two ways: either by restoring the healthy action of the parts, or by obliterating the cavity of the tunica vaginalis. For this purpose, six different modes of operating have been employed. The temporary irritation of the tunica vaginalis by the canula or by a bougie; the introduction of a seton; the excision of the tunica vaginalis; the application of caustic; the injection of a stimulating fluid into the cavity of the tunica vaginalis; or a longitudinal division of the tunica vaginalis through its whole length. The first four methods are now almost universally abandoned. I shall therefore confine my remarks to a comparison between the merits of the cure by injection, and the cure by incision. But, whichever method is preferred, it is desirable to operate before the hydrocele has attained a large size; since, when the hydrocele is very large, the inflammation, by spreading over a more extensive surface, produces more violent symptoms. To avoid this inconvenience, it is usual to evacuate the fluid, watch the progress of the subsequent collection, and, when the hydrocele has attained a convenient size, proceed to perform the radical cure.

"The cure by incision is by far the most ancient, and the most generally employed; the cure by injection has been more recently introduced. Between sixty and seventy years ago, Mr. Sabatier, in the Memoirs of the Academy of Surgery of Paris, published an excellent dissertation on the cure of hydrocele, explaining particularly the cure by injection. About twenty years after, Sir James Earle published an essay, strongly recommending the cure by injection. His recommendation produced a powerful impression on the minds of the British surgeons, so that the cure by injection became the favourite operation. It has the advantage of being more easily performed, and of subjecting the patient to a shorter confinement. But, though the consecutive symptoms are in general more mild, yet a greater proportion of deaths happen in consequence of the cure by injection than of the cure by incision.* It is likewise more uncertain as to its efficacy, as the hydrocele sometimes returns more than once. The possibility of these frequent returns affords a proof that the cavity of the tunica vaginalis is not obliterated. Indeed, the frequency of the secondary effusion, immediately after the operation, leads to the same conclusion. The cure by injection, therefore, must depend upon a change in the action of the parts, not upon an obliteration of the vaginal cavity. This opinion has the support of Mr. B. Bell and Mr. Ramsden.

"The cure by incision, when properly conducted, accomplishes the complete obliteration of the vaginal cavity, which renders a relapse impossible. A gentleman who had the cure by injection performed without success, submitted afterwards to the cure by incision, and declared that the cure by injection was the more painful of the two. I have paid great attention to the subject, with the result of finding my confidence in the certainty and permanence of the cure by injection gradually abate." (P. 126.)

Hernia humoralis, and other ordinary affections, we shall pass without especial notice, as the matter collected is less interesting than on the subject of "Wasting of the Testicles." Before, however, coming to this topic, we must, as an introduction, make room for the following observations on a

"Very interesting sympathetic affection of the testicles (which) occurs in certain cases of cynanche parotidea,† when the pain and swelling abate suddenly, and the affection is transferred directly to the brain, producing convulsions and other dangerous symptoms,

* "As the introduction of a stimulating fluid into the cellular membrane of the scrotum excites a high degree of inflammation, which occasionally proves fatal, the surgeon should be on his guard to avoid this accident by a careful management of the trocar and canula.

† "'Mumps,' in England; 'Branks,' in Scotland."

which occasionally prove fatal. This translation of the secondary affection to an organ of a different class from the one primarily affected, is a very singular deviation from the laws which regulate vicarious affections. Another remarkable peculiarity of this affection is the injurious effects of free evacuations, a practice naturally applied to a case characterized by all the symptoms of active inflammation. Yet it seems a fact well established by those who have had experience in this disease, that the copious detraction of blood brings on those dangerous attacks upon the brain, which admit of relief only by a discharge from one of the organs originally affected. This practice, with the most satisfactory result of relieving the brain from oppression, has been put directly to the test of experiment, by the application of blisters behind the ear, and to the region of the parotid gland. Blisters, indeed, have never, so far as I know, been applied to the testicles, though, from the striking analogy of circumstances, there is great encouragement to try the practice: for the spontaneous resolution of this sympathetic affection of the testicles is accomplished by a copious discharge from the surface of the scrotum; while the suppression of this discharge, either by exposure to cold, or by the application of repellent medicines, induces a translation of the attack to the brain, accompanied by the usual disastrous consequences.

"Besides the above peculiarities, there is a distressing tendency in this sympathetic affection to cause a decay of the testicle. In these unfortunate cases, the decrease of the swelling does not stop when the testicle has been reduced to its natural size, but continues uninterruptedly till the substance of the testicle is completely wasted, nothing remaining but an empty bag, very sensible to pressure, or to any kind of irritation. There are very few cases in which there is only a partial reduction of size. I recollect but one instance of this variety.* M. Richter gives a very curious history of a kind of rheumatic swelling of the testicle, in which the cure was effected by the swelling subsiding below the natural size of the testicle; which, however, afterwards regained its healthy size. But this is a recovery which the patient has little reason to expect. The only well-authenticated case of this is given by Saviard, who, in performing an operation for the radical cure of hydrocele, found the testicle so completely shrunk as to be concealed between the folds of the tunica vaginalis. Upon the cure of the hydrocele, however, the testicle regained its original size." (P. 152.)

Wasting of the testicles, however, is occasionally met with, not as a symptom of any other cognizable disease, but as an idiopathic affection of the organ itself.

"Fortunately," however, (as our author observes,) "the decay of the testicle is not a disease of frequent occurrence, so that the information on the subject lies scattered over the works of surgical

* "Dr. Hamilton's paper upon Mumps."

authors, few of whom have ever seen more than two or three cases of the disease. Baron Larrey is the only person I know who has had practice in it upon anything like an extensive scale, and his account of the disease is exceedingly interesting. After the return of the army from the Egyptian expedition, many soldiers complained of the disappearance of the testicles, without any venereal affection. The testicles lost their sensibility, became soft, diminished gradually in size, and seemed to be dried up. The attack, in general, began in one testicle at a time. The patient did not perceive this decay till the testicle was reduced to a very small size; it approached the inguinal canal, and was about the shape and size of a horsebean. It was indolent, and of a firm consistence. The spermatic cord itself diminished in size, and partook of the atrophy. When both testicles were affected, the patient was deprived of the faculty of procreation, of which he was apprised by the absence of all desire, and by the laxity of the parts of generation. This loss influences all the interior organs. The inferior extremities become lean, and totter under them; the countenance becomes discoloured, the beard thin, the stomach loses its tone, the digestion is impaired, and the intellectual faculties deranged. Several soldiers with this infirmity were invalided.

"This complaint is ascribed to the excessive heat of the climate, the fatigues and privations of war, and, above all, to the use of spirits made from dates, in which different species of Solanum were infused. The ancients are said to have procured the atrophy of the testicles by the continued application to the scrotum of the inspissated juice of hemlock.

"When the atrophy is complete, art does not offer any resource; but, at its commencement, the distressing consequences may be prevented by the use of vapour-baths, dry friction over the surface of the body, urtication of the thighs, refreshing stomachic remedies, and good diet. A person may be secured against this accident by abstaining from the immoderate use of women and spirituous liquors. Since the return from Egypt, Larrey had occasion to treat this malady in many soldiers of the imperial guard, who brought it upon themselves by like excesses. In one person, this malady had, in a very short time, attained an extreme degree of malignity, insomuch as to make both testicles disappear almost entirely. The patient, who heretofore was of a robust constitution, with a thick beard and prominent features, lost all character of virility, and presented the appearance of an effeminate being; his beard was thin, his voice exceedingly feeble and shrill; his genitals without action, and incapable of generating. All means of cure proved ineffectual.

"Similar symptoms have been produced by deep wounds upon the nape of the neck." (P. 156.)

Will phrenology throw any light upon this latter consequence? Will deep wounds in the neighbourhood of the cerebellum impair its presumed functions in regulating the sexual desires, and, of course, the normal state of the subservient generative organs?

"Excessive venery is represented as an exciting cause of this atrophy; while, on the contrary, abstinence from all connexion with women, or uninterrupted continence, has been supposed to produce the same effect. There is nothing inconsistent with the laws of nature, in two opposite extremes destroying the functions of an organ; and this may possibly obtain in the case of the testicles, though the evidence of the fact is not, so far as I know, established upon a sufficiently extensive induction. Supposing it true, the case should not be so hopeless as the other variety, since, by encouraging the patient to employ the parts in discharging their natural function, if the disease be not confirmed, there is a probable prospect of recovery. In the case given by Saviard, the shrinking of the testicles was ascribed to the pressure of the fluid, and therefore did not indicate any radical defect in the testicle. In cases of hydrocele in which the testicle undergoes any change, it is usually that of enlargement. One other case of the diminution of the testicle in hydrocele is recorded, in which the surgeon removed the testicle, supposing it useless and irrecoverable. His practice, in this respect, was precipitate, since the result of Saviard's case gave sufficient encouragement to expect a favourable termination; at least, it would have been prudent to have watched the progress of the case with patience. The rashness of the surgeon in this instance demonstrates the advantage derived from an extensive and intimate acquaintance with the facts recorded by our predecessors, as this knowledge may enable us to treat a case judiciously, which otherwise might be regarded as quite new, and without any previous experience to regulate our practice.

"There is a solitary case, by Dr. Greenfield, of a patient with a decayed testicle, cured by the internal and external use of cantharides. The testicle had decreased to the size of a filbert-nut; but, after the cure, it had recovered its natural magnitude.

"The decay of both testicles is one of those melancholy catastrophes which makes a deep and indelible impression upon the mind of the sufferer. Unfortunately, too, it is one of those cases in which the interposition of art has not hitherto been able to afford any effectual relief; nor is the prospect of discovering any method of cure very flattering, as the cases occur too rarely to afford an opportunity of investigating the subject fully in all its bearings, so as to ascertain which mode of practice is the best. At present, therefore, we must regard decay of the testicles to be a disease beyond the power of art to remove." (P. 163.)

"The testicle has likewise been found completely wasted, after the termination of a tedious severe fever. In this case, there is not any intimation of the approaching evil given by previous alarming symptoms; it is accidentally discovered. I have had an opportunity to witness this form of the complaint, but do not know its nature." (P. 170.)

The remaining portion of the volume is occupied with observations on Neuralgic Affections of the Testicles, on Fungus Hæmatodes, and various other malignant and non-malignant changes which occur in these organs; and the work concludes with a short dissertation on the Diseases of the Spermatic Cord. Our notice, however, has extended to a greater length than we purposed, and therefore we abstain from making any further quotations, and close our analysis by recommending the work to the perusal of our younger brethren, especially students, who will find much valuable information condensed into a small compass, and detailed in concise terms.

---

*A Treatise on some Nervous Disorders; being chiefly intended to illustrate those Varieties which simulate Structural Disease.* By Edwin Lee, Member of the Royal College of Surgeons; formerly House-Surgeon to St. George's Hospital.—8vo. pp. 152. Burgess and Hill, London.

We have no doubt that every practitioner of mature age and experience must have met with cases of hysteria (as they are called), or of anomalous nervous maladies, particularly in young females, in which, upon a retrospection of the opinions he formed at the time of their nature and appropriate treatment, he confesses to himself that he was mistaken, and that he acted upon the principle of the existence of some positive structural disease, when, in fact, there was nothing more than general derangement of the nervous system. In truth, one of the most difficult duties that the medical practitioner has to perform, is to discriminate purely nervous disease from structural disease; and it is no less true that this duty is as important as it is perplexing; for, in most instances, when the subjects of nervous diseases are treated upon the presumption of their labouring under some local disease of structure, the treatment that is adopted not only fails to give relief, but very generally adds to the sufferings of the patient, and renders the diagnosis still more difficult than before the patient had submitted to medical interference at all. If it were our present purpose to write an essay, instead of to make an analysis of the work before us, we could detail very many cases, in which nervous and hysterical young females have been bled, and purged, and treated by strict antiphlogistic means, until their health was irremediably ruined, upon the mistaken supposition of their labouring under local inflammatory disease. The frequent occurrence of such cases has, within the last few years, led to their being more strictly investigated; and, if we cannot yet venture to declare that their true pathology is made clear, we have at least made one great step towards improvement, by having arrived at sufficient knowledge of the subject to enable us to determine, in most instances, with care and attention, that they are not really the diseases of which they so often assume the appearance. Many well-known writers have recently devoted their especial attention to this subject, and, amongst others, we may particularly mention Dr. MARSHALL HALL.* The medical periodicals also teem with cases of nervous diseases simulating structural disease.

In spite, however, of all the labour that has been bestowed upon the subject, much yet remains to be done, before we can flatter ourselves that we know enough of it to act with the confidence which arises only from certain and established principles. We are therefore grateful for any additional information that comes from such respectable authority as Mr. LEE, who, although he addresses himself more particularly to the junior members of the profession, may fairly claim the attention of old and experienced practitioners. He states that his attention was originally drawn to "cases of puzzling and intractable nervous disorder," by the observations made on them by Mr. Brodie, in his Lectures, and he has been induced to publish the present work, from having since witnessed, in several instances, the injurious consequences of nervous disorder being mistaken for inflammatory and organic disease.

Mr. Lee commences with a few observations on the nervous system of men and animals, and next enters into a brief inquiry of nervous disorders in general. Derangement of the functions of the nervous system is prevalent in proportion to the degree of susceptibility of the brain to impressions produced on it by external agents, or by the operations of the mind.

"This cerebral susceptibility rarely exists in a high degree while man continues in a low state of civilization. In proportion, however, to the increased exercise of the intellectual faculties, and to the progress made in the luxury and refinement of civilized nations, does the nervous system become more sensible to pleasing and painful impressions: the causes of nervous excitement become more multiplied, and a high degree of sensibility is engendered; which, while it enhances many of the enjoyments of life, at the same time predisposes to numerous diseases, from which the barbarian and the labouring man, occupied in his daily routine of mechanical employment, are exempt. The Romans, under the republic, enjoyed an exemption from nervous disorders, which forms a striking contrast

* Commentaries on some of the more important of the Diseases of Females. —Longman, 1827.

with their prevalence among the same people in later times. In this country these complaints prevail, at the present day, to an extent unknown at any former period, or in any other nation." (P. 22.)

It cannot be doubted, by any person who draws his conclusion from an attentive examination of different classes of society, that the modern system of education, which tends to the cultivation of the cerebral faculties in a degree disproportioned to the exercise of the bodily powers, is one of the chief causes of the nervous susceptibility becoming excessively developed; the prejudicial effects of which are more evident after puberty, and during the succeeding years of life, when the mental sensibilities are more directly called into action. Civic life developes the nervous temperament and predisposition to disorder in a much greater degree than a residence in the country.

"Women, in consequence of the greater delicacy of their physical organization, and the high degree of nervous sensibility with which they are endowed, joined to their more sedentary mode of life, are easily and more strongly affected by agreeable or painful impressions; and, consequently, are much more subject to nervous diseases than men, who are comparatively exempt from various complaints to which the female sex are liable; but who, as life advances, become more subject to mental and nervous diseases, in consequence of their being more exposed to numerous sources of cerebral excitement, in the worry and turmoil of the world." (P. 25.)

"The influence of moral causes in the production of nervous disorder has not been hitherto sufficiently considered; hence this disorder is often attributed either to the effects which it occasions, or to any disease with which it may happen to co-exist. It is only in cases of mental aberration that due weight is ascribed to these causes in producing the disease. M. Georget, who has ably exposed the fallacy of some of the theories which refer nervous disorders exclusively to irritation or derangement of other organs than the brain, says, in alluding to this subject, 'If the stomach be irritated by improper food, many diseases may be occasioned; in like manner, moral causes may produce disorder of the sensitive and intellectual functions. The only occasions on which importance is attached to disturbance in the functions of the brain is when there is complete derangement; an individual may have insomnia, cephalagia, moral, intellectual, or muscular weakness, but if he is able to reason and follow certain ideas, it is said that the brain performs its functions healthily; but, on the other hand, if an individual experience loss of appetite and slight distaste for food, he is considered to be labouring under gastric disorder.'*

"Persons suffering from various nervous disorders frequently live to an advanced age, and retain an appearance of health, which would be incompatible with the existence of long continued disease of the

* "De la Physiologie du Système Nerveux."—Paris.

parts to which the symptoms are referred. When disease or functional derangement of an organ remote from the brain co-exists with nervous disorder, it can generally be recognised by its characteristic symptoms; and, though rarely the original cause of the cerebral affection, it may react on the brain, so as to keep up and aggravate the nervous symptoms: where a high degree of susceptibility exists, it also frequently proves an exciting cause of relapses." (P. 29.)

Nervous disorders, for the most part, are not accompanied by fever, nor followed by serious consequences. They vary greatly in their symptoms and progress, and are often of long duration; the symptoms being either constantly present, or recurring at regular or irregular periods. Some are productive of much suffering; while others do not occasion pain, and are felt merely as an inconvenience. In some cases, the symptoms frequently shift their situation, affecting various parts simultaneously, or in succession; at other times, they are concentrated towards some part in particular. Although, in general, capable of being greatly alleviated by medical treatment, they sometimes resist all the efforts of art, and either cease spontaneously, or become merged into some other disease.

The general observations Mr. Lee offers upon Hysteria are correct, as far as they go; he does not, indeed, profess to enter very deeply into the subject. He is opposed to the opinion of those, and that justly, who ascribe all hysterical complaints to some uterine derangement, and he thinks that the evidence that hysteria is essentially a disorder of the brain may be deduced from observation of its causes, symptoms, and of the remedial measures which are productive of the greatest benefit.

"In all cases, pain or other unpleasant sensations are referred to the head; the faculties of the intellect, of sensation, and voluntary motion, are more or less impaired. In almost every instance, the disease is induced by the agency of mental or moral impressions, acting either as predisposing, or as exciting causes; and, in the majority of cases, these are the sole causes which operate in producing the disease, the disturbance of the bodily functions being a consequence of the cerebral disorder. In those cases where hysteria is occasioned by local irritation of particular organs, the cause becomes in general apparent, and the disease usually subsides on its removal, as the following cases exemplify:

"'A young woman, æt. 26, in whom menstruation is always performed with difficulty, and scanty in quantity, being by no means proportioned to the quantity of blood circulating in the system, a plethoric state is occasioned. The day previous, or on the day of the appearance of the menses, she is generally attacked by headach, loss of appetite, cramps and pains in the epigastric and hypogastric regions, with sense of weight in the latter part; the legs and arms are debilitated to such a degree that she cannot sustain herself, she sheds tears, and is attacked by convulsions. On recovering, she is weakened and depressed, feels general indisposition for some hours, and sometimes experiences great pain until the menstrual secretion is established. When this has continued two or three days without intermission, she is entirely relieved, and recovers her health and strength until the following monthly period, when similar symptoms recur. Bleeding, leeches, baths, vegetable diet, relieve the patient, without curing her entirely."

"'A girl, æt. sixteen, in whom menstruation was regularly performed, was suddenly seized with convulsive attacks, which returned two or three times a week; she was aware an hour previous to the attack of its being about to recur. It commenced with slight shivering, and a sensation of cold vapor rising from the abdomen to the head; then succeeded loss of consciousness, with convulsive movements of the limbs. After trying various antispasmodic remedies without effect, a purgative was administered, which occasioned copious alvine evacuations, and the passage of two lumbrici; several others were subsequently passed, after which the patient was cured, and had no recurrence.'

"The remedies which are most effectual in mitigating and removing the symptoms, are those which have an immediate action on the nervous system. Such direct effects as are witnessed from the influence of the mind in the prevention of an attack, and on the progress and duration of hysteria, could only occur in a disease of cerebral origin." (P. 57.)

Mr. Lee passes much too rapidly over Epilepsy and Chorea, and we do not discover, in his comments upon these subjects, anything that could profitably arrest our attention, or be worthy our readers' notice.

In the second part, Mr. Lee treats on some special affections of voluntary motion and sensation, and on hypochondriasis.

*Deranged Muscular Action, depending on Cerebral Excitement.* "Fixed contraction of various muscles may be occasioned by the operation of the above-named causes, or it may supervene on some other form of nervous disorder. The degree of contraction varies according to the nature of the part implicated and other circumstances, it is sometimes so strong as to require the employment of much force to overcome the resistance. The efforts of the patient to overcome the rigidity are in some cases unavailing, but generally the patient retains more or less power of moving the part.

"The muscles of the superior extremity appear to be more frequently the seat of fixed contraction than those of the inferior extremity. The flexors of the elbow are often affected, this joint being maintained in a state of flexion or semiflexion while the patient is awake; but if the attention can be abstracted, the part will often become partially relaxed, and may be moved with comparative facility. The joint may sometimes be easily extended, but reverts to the flexed condition as soon as the extending force is withdrawn; at other times, the contraction is attended with pain, which any attempts at extension aggravate. In some cases, the skin of the part is morbidly sensitive to the touch. The fingers are also frequently contracted, the hand being held firmly clenched, particularly when attempts are made to open it, or when the patient's attention is otherwise directed to the complaint. This state is not unfrequently combined with contraction of the flexors of the elbow, or with other nervous symptoms.

"The muscles which raise the lower jaw are occasionally implicated, producing lock jaw more or less complete. When the muscles are so rigidly contracted as to prevent the introduction of substances into the mouth, considerable embarrassment is occasioned; no apprehension need however be entertained of the patient suffering materially from hunger or thirst, as when these sensations require to be allayed, sufficient relaxation will take place to allow liquids to be introduced. Wry-neck is sometimes produced from the muscles on one side of the neck becoming the seat of the contraction. Other parts of the body are also liable to this affection.

"The disorder is not in general of long duration, but relapses not unfrequently occur. In all its varieties, the affected parts become relaxed during sleep, and may then be freely moved. This circumstance will serve to distinguish the more obstinate cases from similar complaints of a purely local and more permanent character." (P. 73.)

The following case is given.

"An unmarried female, æt. twenty, was admitted into St. George's Hospital, in July 1827, having, two months previously, fallen and hurt her left elbow and hip. Considerable pain and discoloration of the elbow were caused by the accident, but subsided after the employment of a liniment. When received into the hospital, the elbow-joint was in a state of semiflexion, and the fingers and thumb firmly closed. While the patient was awake, manual attempts to overcome the contraction caused a kind of hysteric paroxysm. She complained of pain extending from the elbow to the wrist: this was aggravated by moving the forearm, and by lightly pinching up or tapping the skin. The sensibility of the skin in other parts of the body was also morbidly increased, but her general health was not impaired. Mr. Brodie, whose patient she was, prescribed the application of the spirit lotion of the hospital to the elbow, and the following medicine: Tinct. Valer., Ammon., Vini Aloes *aa* ʒi. sextâ quâque horâ ex aquâ.

"The patient feeling relieved by these means, they were continued, with the occasional employment of the shower-bath, for about a month; at the expiration of which period, the pain having entirely subsided, and the patient having regained the use of the elbow and hand, (the contraction recurring only for a short time at distant intervals,) she was placed on the out-patients' list." (P. 77.)

The case next related occurred in an hospital at Florence, and is a good illustration of the mistaken views which even experienced practitioners not unfrequently take of nervous disorders.

"Dec. 10, 1830. Three months ago, a girl, æt. seventeen, in whom menstruation was occasionally irregularly performed, but healthy in other respects, on descending into a close cellar, fainted, and fell to the ground. In falling, she struck her neck against some projecting body; abscess formed in the situation of the injury, was opened, and healed at the expiration of six weeks. Some days before her admission to the hospital, she lost the use of her left arm, and, shortly after, that of the left leg. The extremities of the right side subsequently became paralytic, and she was brought to the hospital in this state in the beginning of November. The intellect, the functions of respiration and digestion, continued unimpaired, as did those of the detrusor urinæ and sphincter ani muscles. The case was considered to be inflammation of the spinal marrow. Repeated bleeding, the application of leeches and blisters along the spine, low diet, the exhibition of strychnine, and the formation of a sore by caustic in the situation of the previous abscess, produced no amelioration.

"A fortnight ago, she suddenly heard of the death of a near relation; and from that time constant movements of the limbs succeeded to the state of paralysis in which they had previously lain. These movements have continued ever since; the arms are incessantly beating against the breast, the thighs and legs alternately bent and extended with violence. Though pale, her countenance does not indicate the existence of organic disease; the intellectual and vital operations are not impaired; she answers questions readily; the tongue is clean, the pulse weak. The prognosis delivered by her physician is unfavourable. She takes no medicine, but leeches are occasionally applied along the spine.

"I ascertained that the motions of the limbs, though constant during the day, did not prevent her from sleeping at night, at which period they ceased. They were more violent when any one approached the bed, and conversed with her, but, when she was not conscious of being observed, their violence was lessened, and they occasionally ceased altogether for a few seconds. The patient had the power of so far interrupting the movements as to put her hand to her head, when directed so to do, and to point to anything she wanted.

"From a consideration of the peculiarities of the case, and of its long duration, I was led to infer that, though the paralysis might have been occasioned by some irritation of the nerves, consequent on the healing of the abscess, the present symptoms did not indicate disease of the spinal marrow; that the complaint was essentially nervous, having great analogy with chorea, the motions of the limbs being, as in that disease, in some degree kept up by habit. This view of the case I communicated to her physician, who did me the honour to ask my opinion.

"Dec. 24th. The depletory measures have been discontinued, and the quantity of food increased, since the 14th. The patient has had, during the last two days, several hysterical symptoms; such as tremulous motions of the eyelids, loss of voice, occasional fits of laughter. The movements of the limbs are less violent, and at times cease altogether; she sleeps well, and her appetite is good.

"Dec. 30th. The patient, having been allowed a more full diet, is much improved in appearance; the movements are now almost entirely confined to the hands, and cease if her attention can be drawn off from her complaint.

"From this time she recovered rapidly, and was dismissed from the hospital in January." (P. 83.)

*Deranged Muscular Action, dependent on Cerebral Torpor, or Debility.* Under this title, Mr. Lee makes some good practical remarks on aphonia, retention of urine, ptosis, and paralytic affection of the extremities. The following curious case of paralysis of the lower extremities, of seven years' duration, is taken from Dr. Bright's Reports of Medical Cases.

"'A delicate young woman, æt. twenty-four, was admitted into Guy's Hospital, Oct. 25, 1826. She had so completely lost the use of her lower extremities, that she was unable to walk a step without support. It appeared that this had been her condition for the last seven years. The catamenia had been obstructed about the time this complaint first came on, and for two years had not made their appearance. No disease could be detected on careful examination of the spine. She occasionally complained of pain in her temples, and the bowels were constipated. The medicines ordered, were directed entirely to strengthen her general condition, by bringing the stomach and bowels into better action, and the employment of counter irritation, both by ointment of tartarized antimony, and repeated blisters to the loins. For a long time, very little improvement took place, the catamenia, retained in sparing quantity, and attended by great dysmenorrhœa, but at length, when she had been seven or eight months under treatment, she became rapidly well, and left the hospital walking as if she had never been ill." (P. 99.)

*Nervous Disorders affecting Sensation.* The perception of impressions may be morbidly acute, or perverted, or it may be obtuse, or partially suspended. Moral causes operate greatly in effecting the perception of sensation, as do also over-exertion of the sensitive and intellectual faculties, visceral irritations, and general debility of the system. In the treatment of these affections, Mr. L. correctly states, that energetic depletory measures will not frequently be required. The mental and moral management of the patient will influence greatly the course and duration of the complaint.

"Increased sensibility of any part of the surface may frequently be occasioned by lightly pressing or pinching-up the integuments, from the attention of the patient being directed to the manipulation. The pain and tenderness to the touch, which are the leading symptoms, are however mostly circumscribed to particular parts; and hence resemble inflammatory, and other diseases of organs subjacent to that part of the skin to which the symptoms are referred. In consequence of the nervous affection being mistaken for structural disease, patients are frequently subjected to much injury from an improper mode of treatment. Some in whom the symptoms were referred to the back, have been confined to the horizontal position, and caustic issues kept open for months together, from the patient being supposed to be afflicted with spinal disease; others have been subjected to energetic depletory measures, under the supposition of inflammation existing in some important part. These diagnostic errors, even if productive of no other harm, keep up the disease, by increasing the nervous irritability of the system, while the treatment adopted tends to debilitate the patient, who is confirmed in the idea, from the length of time the complaint exists, unmitigated by the remedial means employed, that she is labouring under an intractable and incurable disease; hence there is less probability of altering the morbid perception of sensation in which the disorder consists, and unless some circumstance occur to divert the mind from the habitual consideration of the complaint, it may continue for almost an indefinite period, the remedies employed being only productive of palliative effects, until recovery spontaneously takes place, or until from long confinement and impaired general health some organic disease be induced.

"It is sometimes difficult to discriminate the nervous disorder from organic disease; the temperament of the patient, the course of the disorder, its peculiar symptoms, and the absence of febrile disturbance, will in most cases serve to distinguish it. It must however be borne in mind, that nervous affections are sometimes superadded to structural disease, which then acts as their exciting cause. The definite character of the symptoms of structural disease will in general make its existence evident." (P. 116.)

Different parts of the body may have their sensibility greatly increased, without structural disease. The knee is, perhaps, most frequently the seat of this nervous affection; and very often the disorder affects the breast in young women. It is often combined with tumor of the mamma, occasioning great alarm amongst the patient's friends, who are apt to regard it as of a schirrous nature. Sir A. Cooper describes this affection by the name of irritable tumor of the breast, and, as examples of it, Mr. Lee gives two cases.

"Case I. A young girl, æt. seventeen, was admitted into St. George's Hospital, on the 20th August, 1829, with painful tumor of the left breast. The tumor, hard, and of the size of a large filbert, had existed three years, but only became painful after an attack of fever, six months previous to her admission. The pain was not constant, but came on at intervals, more or less distant, being always increased by mental agitation. The skin covering the breast and surrounding parts was exquisitely sensitive when touched, the pain remaining several hours after the manual examination. Leeches and cold lotions were employed, without advantage. The patient's general health was good, and menstruation regular. She was ordered a belladonna plaster to the painful breast, and the following draught every six hours. ℞. Mist. Camphoræ ℥i., Decoct. Aloes comp. ʒiij., Tinct. Humuli ʒi. These measures relieved the pain and morbid sensibility of the skin; they were continued for about three weeks, when these symptoms having entirely subsided, and the tumor being somewhat diminished in size, the patient was discharged.

"Case II. A young girl, æt. twelve, was received into St. George's Hospital, in October 1829, having a tumor, of the size of a walnut, in the left breast, which came on attended by fever two years previously, and attained the size of a small orange, but had since gradually subsided. At the time of her admission, the tumor was occasionally painful; the pain recurring at irregular periods, extending down the arm of the same side, and lasting about ten minutes each time. The skin of the breast and its neighbourhood was morbidly sensitive to the touch. These symptoms had, however, existed only since the month of June. The general health was good, and the sleep undisturbed. The employment of antiphlogistic means had not been attended with advantage.

"She was ordered to take, every morning and noon, Vini Ferri et Vini Aloes *aa* ʒi. ex aquâ; and the following embrocation to be rubbed on the breast twice a-day: ℞. Linim. Camph. ʒiss., Tinct. Opii iij. M. fiat embrocatio. At the expiration of a week, the symptoms were greatly alleviated, and she was transferred to the out-patient's list." (P. 134.)

Pain and preternatural sensibility of the skin often affect the back, especially some part along the course of the spine, and the disorder is liable to be mistaken for spinal disease, if attention be not paid to the diagnostic marks. This, Mr. L. remarks, is the more likely to happen as the affection is frequently attended by debility, more apparent than real, of the lower extremities, particularly if the patient have been long confined to the recumbent position.

"The pain is sometimes diffused over a large extent of surface, but is more frequently limited to some point of the spine, and is aggravated by pressing on the part, or by lightly patting and pinching-up the skin. The morbid sensibility is not however confined to the part to which the pain is referred, but is in general more extensively diffused over the back, and occasionally affects at the same time other parts of the body, the patient complaining of pain being induced by the slightest touch. The pain is not always confined to the same point, but shifts its situation, and is often absent altogether for a short period. The patient sometimes complains of weakness in the loins when attempting to stand or sit upright. The symptoms, however, vary at different times, the patient being on some days better, on others worse. The general health is not materially impaired, and the sleep is not disturbed by the complaint, which is occasionally of long duration, without any increase in the severity of the symptoms.

"The diagnosis between this disorder and spinal disease is easy. In spinal disease, the pain, when present, is fixed to one point, and requires pretty firm pressure on the part to increase it; while, in the nervous affection, it is more variable, and shifts its situation. As disease of the spine advances, the patient's appearance becomes altered, fever supervenes in most cases, the limbs become paralytic, abscess frequently shews itself in the loins or groin, and projection of one or more of the spinous processes will be perceptible when the dorsal vertebræ are affected. The morbid sensibility of the skin to the touch is not, in general, present in spinal disease: I have however seen in one case this symptom co-exist with disease of the spine, in which there was manifest projection of the spinous processes of two dorsal vertebræ, and a paralytic state of the lower limbs; the skin of the back, breast, and abdomen, was acutely sensitive. The patient was a female who had formerly been subject to hysteria. (P. 136.)

"A young woman, whose countenance indicated good health, was admitted into St. George's Hospital, in the beginning of April 1829, complaining of acute pain in the small of the back, which had existed, with occasional intermissions of three or four days, for three years and a half. It was sometimes transferred to other parts of the back, and was aggravated at her monthly periods; menstruation was otherwise easy and regular. She had been salivated by mercury, and confined to the horizontal position for twelve months, during part of which time a caustic issue was kept open on either side of the spine.

"Though her general health was unimpaired, she was unable to stand without support. Pressure on any part of the spine occasioned pain. The skin covering the spine, and in other parts of the body, was morbidly sensitive to the touch. She could move the legs freely when lying or sitting down, and her sleep was undisturbed. She was ordered to take, three times a-day, Spirit. Ammon. comp. ♏x., Misturæ Camph. ʒx.; and was allowed to get about the ward on crutches.

"At the expiration of a fortnight, she considered herself to be better, and had gained more strength in the legs. The medicine was continued, and a large blister applied to the loins.

"In the beginning of May, the pain and sensibility of the skin were greatly mitigated; the patient made great objection to the blister, which she requested might not be repeated: a fresh blister was nevertheless applied, and the same medicine continued.

"A third blister was applied after a few days; and, in the middle of May, the patient was dismissed the hospital cured, being able to walk without any other assistance than a stick." (P. 139.)

